# Expression of Neurotrophin-3 and trkC following Focal Cerebral Ischemia in Adult Rat Brain with Treadmill Exercise

**DOI:** 10.1155/2017/9248542

**Published:** 2017-09-06

**Authors:** Jin-Young Chung, Min-Wook Kim, Wooseok Im, In Koo Hwang, Moon-Suk Bang, Manho Kim

**Affiliations:** ^1^Department of Veterinary Internal Medicine and Geriatrics, College of Veterinary Medicine, Kangwon National University, Gangwon-do 200-701, Republic of Korea; ^2^Department of Rehabilitation Medicine, College of Medicine, The Catholic University of Korea, Seoul 150-713, Republic of Korea; ^3^Institute of Catholic Integrative Medicine (ICIM), Incheon St. Mary's Hospital, Incheon 403-720, Republic of Korea; ^4^Department of Neurology, Seoul National University Hospital, 101 Daehakro, Chongno-ku, Seoul 03080, Republic of Korea; ^5^Departments of Anatomy and Cell Biology, College of Veterinary Medicine and Research Institute for Veterinary Science, Seoul National University, Seoul 08826, Republic of Korea; ^6^Department of Rehabilitation Medicine, Seoul National University Hospital, 101 Daehakro, Chongno-ku, Seoul 03080, Republic of Korea; ^7^Neuroscience and Protein Metabolism Medical Research Center, Seoul National University, College of Medicine, 101 Daehakro, Chongno-ku, Seoul 03080, Republic of Korea

## Abstract

Neurotrophin-3 (NT-3) is a neurotrophic factor that mainly binds to the tyrosine kinase C (trkC) receptor. NT-3 has been shown to have neuroprotective effects in focal cerebral ischemia. Exercise also has ability to induce functional recovery in focal cerebral ischemia. However, the relationship between NT-3, its receptor trkC, and exercise has not been revealed. In this study, we assessed the expressions of NT-3 and trkC in focal cerebral ischemia. We also assessed the expression of NT-3 and trkC with treadmill exercise in focal cerebral ischemia. The results showed that, in a permanent middle cerebral artery occlusion rat model, exercise increased NT-3 and trkC expression. However, the patterns of expression of NT-3 and trkC at different time points varied. These results suggest that exercise-induced functional recovery in focal cerebral ischemia was related to NT-3 and trkC, but the role on times of NT-3 and trkC differed, although trkC is the receptor kinase for NT-3.

## 1. Introduction

Stroke is the fifth leading cause of death in the United States, with approximately 800,000 people a year having a stroke, or one every 40 seconds [1]. Stroke results in huge health burdens, and the American Heart Association (AHA) suggested that the direct medical costs of stroke will increase by 238% from 2010 to 2030 [2].

Despite numerous trials to ameliorate stroke, there have been no satisfactory outcomes. However, exercise therapy after stroke has long been considered a logical candidate to ameliorate physical disability [3]. Although various studies have investigated behavioral improvements and structural alterations in the brain with postinjury exercise therapy, little is known about the underlying mechanisms [4].

Many studies have also demonstrated that neurotrophic factors play important roles in neuronal survival, proliferation, maturation, and outgrowth in the developing brain and neuroprotective function in mature brain insult [5]. These studies have revealed changes in several neurotrophic factors in the stroke model [6–8], indicating that, in the situation of stroke, exercise therapy can ameliorate the physical disability related to neurotrophic factors.

Neurotrophin-3 (NT-3) is one of the neurotrophic factors, which comprise a family of proteins that includes nerve growth factor (NGF), brain derived neurotrophic factor (BDNF), NT-3, and neurotrophin-4 (NT-4). The neurotrophic factors function via interactions with different members of tyrosine kinase (trk) receptors [9]. Each neurotrophic factor has its own high affinity receptor. NT-3 mainly binds tyrosine kinase C (trkC), which apparently acts as a downstream physiological receptor.

In this study, we investigated the effects of treadmill exercise on the expression of NT-3 and trkC in a rat model of experimental cerebral infarction.

## 2. Materials and Methods

### 2.1. Experimental Design

In this study, we used 59 adult Sprague-Dawley rats. All of them were male with body weight ranging from 275 to 325 g for each rat. Female rats were not included in this study to avoid bias due to sexual difference. Rats were divided into several groups, such as middle cerebral artery occlusion (MCAO) group as well as sham-operation as a control. There were 35 rats in MCAO group and 12 rats as control in number. The MCAO group was further divided into two subgroups with or without exercise in two days. 18 of them had exercise and 17 of them did not have exercise; thus we define these subgroups as “exercise versus nonexercise.” Garcia scale was used to determine the degree of severities. Garcia scale contains six items (spontaneous activity/symmetry of spontaneous movements/symmetry of forelimbs during movement/climbing the wire cage/reaction to touch/response to vibrissae touch). The higher score were the better performance. We measured total score to further define the range of severities. The total score ranged from 3 to 18. We subcategorized into mild group (I, 12–18), moderate group (II, 8–11), or severe group as III with the score of less than 7 (including 7) [6–8]. In addition, in order to explore time-dependent pattern, 12 rats were included from the ischemic-exercise group. Among them, they were scarified every week, for example, at 8, 16, and 23 days following ischemia (*n* = 4 for each of the three groups) Protocols including procedures for use of animals and the care were approved by Catholic University Animal Care Committee according to the compliance guidelines.

### 2.2. Operative Procedures: The Modified Longa's Method

For the experimental focal cerebral infarction model, this method had been used according to what was previously described elsewhere [10]. Initially, 3% isoflurane within 30% O_2_ and 70% N_2_O was used for induction. Isoflurane (1.5%) was used for maintenance. Following anesthesia, the left common carotid artery was exposed through a midline cervical incision. As next step, external carotid artery (showing level at bifurcation as branches) was coagulated. And then, ligation of the pterygopalatine artery was performed with a 5.0 silk suture. Nylon monofilaments (4.0) were used for the occlusion. For occlusion procedure, the monofilament tip was rounded by heating and was inserted into the level of bifurcation site of carotid artery. By advancing 16–18 mm, it can occlude the origin and proximal part of the anterior cerebral artery. We closed the wound with monofilament being in place. Following these procedures, rats were allowed freely to food and water. Rectal temperature was maintained at 37 ± 1°C. We used a thermistor-controlled heating blanket [6–8].

### 2.3. Treadmill Exercise

To further access the effect of exercise, we applied the treadmill (Columbus Instruments, USA). This treadmill exercise was started in two days following the operation, not immediately. In exercise program, on the first exercise day, 10 m/min was given. We increased gradually up to 15 m/min on the second day. On the third and subsequent days, exercise set was 20 m/min. The tilting angle of the exercise table was maintained and set to 0°. This protocol was performed every 30 minutes for 12 days [6–8].

### 2.4. Immunohistochemistry

At day 16, rats were anesthetized first and then sacrificed. For transcardiac perfusion, heparinized saline and then 4% paraformaldehyde in phosphate-buffered saline (PBS) were perfused. Sections were cut at a thickness of 30 *μ*m using a sliding microtome. For blocking, 10% normal goat serum (NGS), 1% bovine serum albumin (BSA), 0.2% Triton X-100, and 1% H_2_O_2_ were used in PBS. Following washing with PBS (×3), anti-NT-3 (1 : 300, Santa Cruz, CA, USA) and anti-trkC (1 : 300, Santa Cruz, CA, USA) antibodies were used in 10% NGS and 1% BSA for overnight at cold room with temperature of 4°C. We used DAB kit (Dako, Carpinteria, CA) for immunoperoxidase labeling. They were evaluated and captured using an BX51 microscope (Olympus, Japan) [6–8].

### 2.5. Immunoblotting

Following anesthesia, the brains were removed from the skull and these were dissected into the right or left hemispheres. For protein extraction, they were placed in 10 volumes of cold homogenization buffer (120 mM NaCl, 50 mM Tris, pH 7.4) with protease inhibitors (Complete Mini, Gibco, Grand Island, NY, USA) being freshly added. Tissue then was homogenized by sonicator. Concentrations of protein were checked by the Bradford method (BioRad, Richmond, CA, USA). By adding the sampling buffer, equal amount of protein, 20 *μ*g, was loaded and separated. Sodium dodecyl sulfate-polyacrylamide gel electrophoresis was used with 10% polyacrylamide and 0.05% bis-acrylamide [11]. By separating proteins on the gels, they were transferred into nitrocellulose membrane. They were probed with anti-NT-3 (1 : 300, Santa Cruz, CA, USA) and anti-trkC (1 : 300, Santa Cruz, CA, USA) as primary antibody. For secondary antibody, Peroxidase anti-rabbit IgG (Vector, PI-1000, 1 : 3000 dilution) was used. As an internal control, anti-*β* tubulin (1 : 300, Santa Cruz, CA, USA) was checked on the sample membrane. We detected signals with enhanced chemiluminescence (Supersignal, Pierce, Rockford, IN, USA), using autoradiogram by exposing 10 to 30 min [6–8].

### 2.6. Statistical Analysis

The Mann–Whitney test and Kruskal-Wallis test were used for groups comparison. We used SPSS (ver. 12.0). *p* < 0.05 were considered to be statistically significant. We triplicated experiments to see whether the findings were replicated or not.

## 3. Results and Discussion

**Figure 1:**
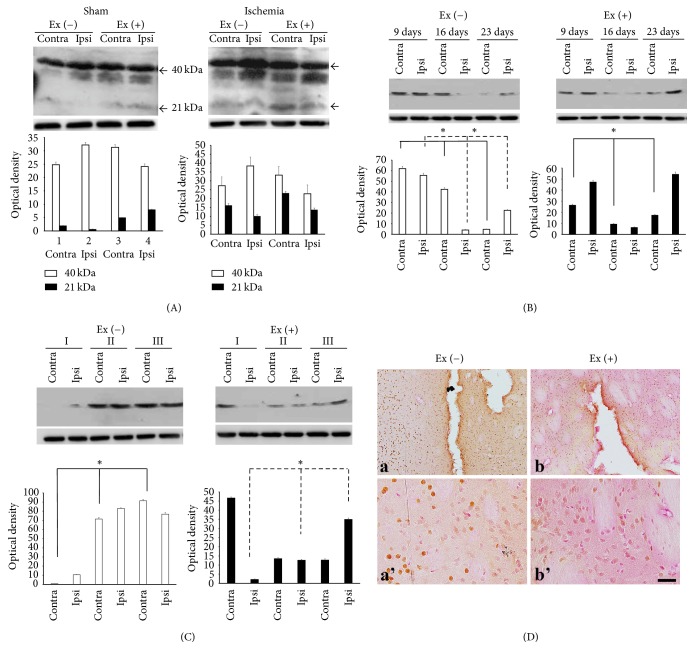
Expression profile of NT-3. (A) There are two forms of NT-3, dimer (40 kDa) and monomer (21 kDa). Exercise in the sham group increased immunoreactivities of the monomer. Exercise in ischemia increased the monomer in the contralateral hemisphere (contra). (B) In the ischemia-nonexercise group, expression of the NT-3 dimer protein decreased from postinfarct day 9 to postinfarct day 23 in the contralateral region. In the ipsilateral region, expression of NT-3 dimer protein was decreased at postinfarct day 9 and then increased at postinfarct day 23. However, in the ischemia-exercise group, expression of the NT-3 dimer protein was decreased at postinfarct day 16 and then increased at postinfarct day 23. (C) The expression of NT-3 dimer protein increased along with severities II and III in the ischemia-nonexercise group. However, expression of NT-3 dimer decreased in the ischemia-exercise group. (D) (a) Immunoreactivity decreased in the core of the infracted region. In contrast, staining increased around the ischemic area. (b) Exercise decreased this contrasting effect. (a') and (b') are magnificent figures of (a) and (b) each. ■ = 25 *μ*m (^*∗*^*p* < 0.05).

NT-3 exists in either a dimer (40 kDa) or a monomer form (21 kDa) protein. Exercise increased the immunoreactivities of monomer protein in both hemispheres of the sham-exercise group. In the ischemia group, exercise led to increased signals in the contralateral hemisphere (Figure 1(A)).

Temporal changes in the NT-3 dimer were observed in the ischemia-nonexercise group. Specifically, these signals decreased from postinfarct day 9 to day 23 in the contralateral region (*p* = 0.027). In the ipsilateral region, expression of the NT-3 dimer decreased at the time point of postinfarct day 9, while enhanced immunoreactivities were observed on postinfarct day 23 (*p* = 0.027). In the ischemia-exercise group, expression of the NT-3 dimer was decreased at postinfarct day 16 but increased again at postinfarct day 23 (contra: *p* = 0.027, ipsi: *p* = 0.050) (Figure 1(B)).

The expression of the NT-3 dimer increased in severities II and III in the ischemia-nonexercise group (contra: *p* = 0.027, ipsi: *p* = 0.050). However, in the ischemia-exercise group, expression of NT-3 dimer protein decreased these signals (contra: *p* = 0.050, ipsi: *p* = 0.027) (Figure 1(C)).

Immunohistochemistry showed that immunoreactivity decreased in the infracted area but increased in and around the infarct core region in the ischemia-nonexercise group. However, exercise appeared to restrict immunoreactivity only in and around the ischemic region (Figure 1(D)).

**Figure 2:**
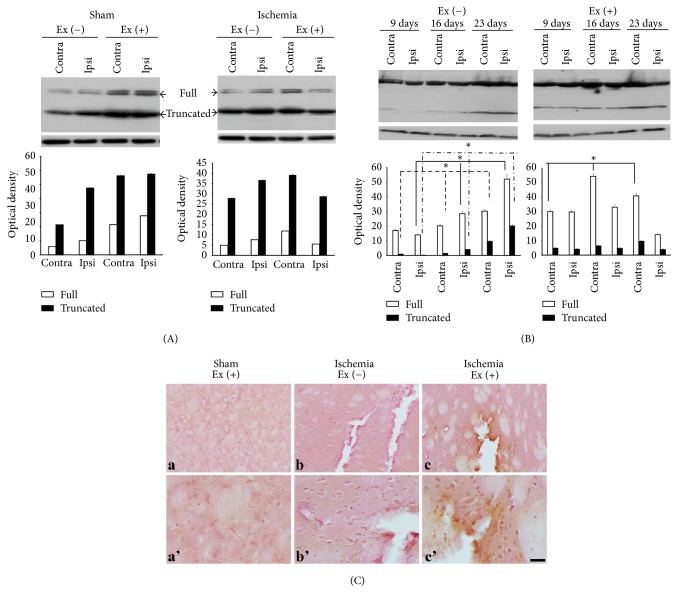
trkC expression profile. (A) There are two forms: full-length (140 kDa) and truncated (90–95 kDa). Ischemia increased both forms, while exercise increased both forms of protein in the bilateral hemispheres, particularly in the contralateral hemisphere (contra) in the ischemic brain. (B) The maximal expression of the two forms was observed at day 23 in the ischemia group. In the exercise group, the full-length form increased with time in the contralateral region, while the expression of the full-length decreased with time in the ipsilateral region. The truncated form was increased at postinfarct day 23 in the contralateral region. (C) (a) In the sham group, exercise itself increased immunoreactivities in both hemispheres, particularly in the vascular structures. (b) Immunoreactivity also increased in the ischemic region. (c) The immunoreactivities were restricted following exercise. (a'), (b'), and (c') are magnificent figures of (a), (b), and (c) each. ■ = 25 *μ*m (^*∗*^*p* < 0.05).

trkC exists either as a full-length (140 kDa) protein or as a truncated (90–95 kDa) protein. Exercise increased the two forms of trkC in the sham group. In the ischemia-nonexercise group, both forms of trkC were increased in the ipsilateral region. In the ischemia-exercise group, these immunoreactivities were decreased in the ischemic ipsilateral region, while they were increased in the contralateral nonischemic region (Figure 2(A)).

Time-dependent patterns showed that both forms of trkC were increased at postinfarct day 23 in the ischemia-nonexercise group. In the ipsilateral region, both forms of trkC increased with time (full: *p* = 0.027, truncated: *p* = 0.027). In the ischemia-exercise group, expression of the full-length form increased with time in the contralateral region (*p* = 0.027). Conversely, expression of the full-length form decreased in the ipsilateral region. The truncated form was increased at 23 days after infarct on the contralateral nonischemic region (Figure 2(B)).

Immunohistochemistry showed that exercise increased immunoreactivity in both hemispheres of the sham group, particularly in the vascular structures, and that exercise also concentrated immunoreactivity around the ischemic region in the ipsilateral hemisphere (Figure 2(C)).

In previous studies, we confirmed the expression of BDNF/trkB, NGF/trkA, and NT-4/trkB following focal cerebral ischemia with exercise. Interestingly, exercise increased the level of BDNF/trkB, NGF/trkA, and NT-4/trkB in the contralateral hemisphere; however the time points were different according to the neurotrophic factors and their receptors [6–8]. Based on these results, we hypothesized that the time points of each neurotrophic factor and receptor are different for neural protection or recovery. For the identification of another neurotrophic factor and its receptor, we observed whether changes in NT-3 and trkC expression in a permanent ischemic middle artery occlusion rat model occurred in response to exercise.

Many studies have been conducted to characterize neurotrophin [12–14]. Among neurotrophins, NT-3 showed two peaks, monomers and dimers, based on elution profiles with western-blot analyses. NT-3 monomers were 100–1000 times less active when compared with dimers in the phosphorylation assay. These data indicated that NT-3 dimers interact far more efficiently than monomeric NT-3 with their receptors [13]. We also confirmed that the expression of NT-3 monomers was less than that of NT-3 dimers in the focal cerebral ischemia. However, although the expression of NT-3 monomer was lower in both the sham and ischemia group, increased tendency for expression of NT-3 monomers was observed after 2 weeks of treadmill exercise.

Previous studies showed that exercise has neuroprotective effect and can increase neurogenesis. The duration and intensity of exercise are important factors promoting the plasticity and enhancement of the brain. Indeed, progressive exercise like walking on a treadmill was sufficient to improve brain function [8, 15, 16]. Neuroprotective effect of NT-3 in the cerebral ischemia was confirmed by exogenous delivery of the NT-3 gene in a focal cerebral ischemia of rat model. However, the mechanism underlying the neuroprotection of NT-3 against focal cerebral ischemia is not completely understood [17]. In this study, exercise increased the expression of NT-3 and focal cerebral ischemia itself increased the expression of NT-3. We can assume that the brain itself expresses NT-3 for neuroprotective effect against injury in the brain and that exercise built up the expression of NT-3. Interestingly, without exercise, ischemia itself increased the expression of NT-3 initially, after which it gradually decreased. However, exercise resulted in the expression of NT-3 being maintained consistently. However, we were unable to determine the mechanism through which exercise changed NT-3 or induce recovery of the focal cerebral ischemia.

Although the mechanism of neuroprotective effect of NT-3 is not completely understood, several studies have shown that NT-3 binds to high affinity receptor trkC, triggers the receptor, and signals cascades. These intracellular signaling pathways modulate gene expression and are responsible for most of the neuroprotective effects related to neuronal growth, survival, and differentiation [17–19].

In this study, we showed that the expression of trkC was also increased by treadmill exercise itself. However, in focal cerebral ischemia, exercise decreased the expression of trkC in ipsilateral region. Moreover, the expression of trkC gradually increased by times without exercise in focal cerebral ischemia. These results are contrary to the expression of NT-3. Exercise increased the expression of trkC, as for NT-3, in the contralateral region, but not in the ipsilateral region in focal cerebral ischemia.

## 4. Conclusions

Overall, the results of this study indicate that exercise induced functional recovery in focal cerebral ischemia and that this effect was related to NT-3 and trkC. However, the roles of NT-3 and trkC likely differed based on the observed differences in the timing of their expression. Therefore, although trkC is the receptor kinase for NT-3, expression profiles of NT-3 and trkC are not directly matched among the time points of exercise-induced functional recovery.
